# Influence of platelet-rich plasma on RANKL and IL-1 immunohistochemical expression in periodontitis-related bone cell proliferation and differentiation

**DOI:** 10.1016/j.sdentj.2024.11.011

**Published:** 2024-12-01

**Authors:** Hana H. Mustafa, Snur M.A. Hassan, Sozan Ali Mohammed, Mardin O. Mohammed, Hadia Karim Zorab, Hardi Fattah Marif

**Affiliations:** a Department of Surgery and Theriogenology, College of Veterinary Medicine, University of Sulaimani, Sulaimani 4601, Iraq; b Department of Anatomy and Histopathology, College of Veterinary Medicine, University of Sulaimani, Sulaimani 4601, Iraq; c Department of Clinic and Internal Medicine, College of Veterinary Medicine, University of Sulaimani, Sulaimani 4601, Iraq

**Keywords:** Bone regeneration, IL-1β, Periodontitis, PRP, RANKL

## Abstract

**Background:**

Platelet-rich plasma (PRP) is utilized as an autologous blood product to encourage bone regeneration. The receptor activator of nuclear factor-NB ligand (RANKL) is a key and central regulator of osteoclast homeostasis. A rat model of experimentally generated periodontitis was used to assess the impact of PRP preparation on the expression of the osteoclastogenic and pro-inflammatory markers respectively; RANKL and IL-1β.

**Material and Methods:**

To induce periodontitis by silk ligature, thirty-six adult male *Sprague Dawleys* rats were used and they were allocated into three equal groups (n = 12): group I consisted of intact periodontal tissue, group II; rat-induced periodontitis without treatment by PRP, and group III of periodontitis **+** 10 µL PRP injection. The rats were sacrificed after both experiment durations (7 and 30 days), and the incisor teeth were fixed and decalcified in HCl for a day and in 10 % EDTA solution for eight weeks at room temperature then samples were processed for H&E stain for bone healing scores and bone cells counting, and the samples were utilized by IHC for detection of both RANKL and IL-1β expression.

**Results:**

The PRP enhanced the process of healing on days 7 and 30 showed (Score 10) vs. the control positive group that had a delay in alveolar bone regarded as (Score 4) significantly (P ≤ 0.05). The PRP group attenuated significantly (P ≤ 0.05) the alveolar bone loss by increasing the number of osteoblasts and declining the proliferation of osteoclast vs. the control positive group that revealed bone destruction due to rising osteoclast proliferation and decreasing the osteoblast proliferation significantly (P ≤ 0.05). PRP inhibited the IL-1β expression (score = 0) vs. the control positive group that showed moderate staining of positive cells detected in both inflammatory cells and endothelium (score = 4). Regarding the RANKL expression, the PRP reduced its expression in vs. the control positive group (score = 4 vs. 12 respectively).

**Conclusion:**

PRP is an anabolic agent that enhances proliferation of osteoblast and inhibit the osteoclast differentiation by downregulation of IL-1β and RANKL.

## 1 Introduction

The gram-negative bacterial infection that causes periodontitis, is one of the most frequent chronic inflammatory responses that may spread into the alveolar bone if the gingiva is not stopped from inducing inflammation. Periodontitis is characterized by the breakdown of alveolar bone and connective tissue as a result of an inflammatory process (Mustafa et al., 2022). It is characterized by elevated expression of several inflammatory mediators and cytokines like TNF-α and IL-1β are important regulators of osteoclastogenesis both in vivo and in vitro, leading to extensive bone loss and osteoclast formation (Nokhbehsaim et al., 2019). These cytokines influence bone remodeling and are essential for the regulation of bone in both physiological and pathological settings (Xu et al., 2023).

Bone resorption and formation work in harmony to maintain the amount of bone mass needed to carry out various tasks. Two specialized cells osteoblasts, which form bone, and osteoclasts, which resorb bone are in charge of this process and are highly coordinated. Numerous hormones, inflammatory mediators, cytokines, and growth factors, in particular, IL-1β control these cells (Salhotra et al., 2020).

RANKL is a membrane-bound or soluble protein that is predominantly formed in osteoblastic lines and stimulated T-cells. Inhibiting osteoclast apoptosis and promoting osteoclast differentiation and activation. Osteoclast activation and differentiation are promoted by RANKL binding to receptors of RANKL that are expressed only on the surface of osteoclasts and their progenitor cells (Chen et al., 2014). Resorption of periodontal bone is accelerated by osteoclasts. The equilibrium between osteoblasts' creation of new bone and osteoclasts' resorption of preexisting bone determines the mass of bone. The main molecule regulating osteoclast activity, recruitment, and differentiation is the nuclear factor-κB ligand (RANKL) receptor activator (RANKL) in combination with its receptor RANKL (Salvi and Lang, 2005).

The term “platelet-rich plasma” describes an autologous blood product with a platelet content above typical physiological parameters, which is created by centrifuging whole blood (Collins et al., 2021, Prokurat et al., 2024). Numerous investigations have demonstrated that PRP enhances osteoblast migration, adhesion, proliferation, and settlement because of high concentrations of growth factors like PDGF, TGF, and IGF, which improve wound healing. In particular, PDGF is a potent stimulant for the chemoattraction of osteoblasts at different stages of differentiation (Lubkowska et al., 2012), TGF-β1 is a regulatory protein involved in bone remodelling and fracture healing (Sarahrudi et al., 2011), and VEGF promotes bone healing by vascular structures and is significant for regulating osteoclasts in the remodeling phase (Hu and Olsen, 2016). The function of platelet-rich plasma (PRP) in bone defects has steadily gained attention nowadays, its impact on RANKL-induced osteoclast differentiation, however, has not yet been thoroughly determined.

Very little study has been done on osteoclasts; most previous studies have concentrated on the impact of PRP on osteoblasts and matrix proliferation, therefore, the current study's objective was to determine how well platelet-rich plasma (PRP) repaired alveolar bone by enhancing osteoblast proliferation and controlling osteoclast through RANKL and IL-1β immunohistochemical analysis.

## 2 Materials and methods

### 2.1 Experimental design

Thirty-six male Sprague Dawley rats weighing between 150 and 180 g were adopted for this study at the seven-week mark. The animals were transferred to the animal house in a veterinary teaching hospital of the University of Sulaimani's College of Veterinary Medicine. Permission 030526, December 11, 2023) authorizes all procedures for the handling, treatment, and sampling of animals at Sulaimani University in Kurdistan, with the approval of the Ethics Committee.

The animals were allowed one week to acclimatize before the trial began. The rooms were maintained at a consistent 22 °C and had 12 h of light and 12 h of darkness. Good ventilation and neat maintenance were features of the room. The rats are provided with standard lab food pellets and are always able to access filtered water in bottles. Every rat is in good physical condition.

Three major groups of rats were randomly assigned to be divided into: Rats in the negative control group (n = 12) had a normal periodontium and had not induced periodontitis; rats in the positive control group (n = 12) had 4/0 Silk suture ligature-induced periodontitis; PRP treatment group (n = 12), rat were underwent periodontitis with silk ligature + PRP therapy. According to experiment duration, each group was split into two replications (n = 6), for 7 days and 30 days of experiment.

#### 2.1.1 Induction of periodontitis

The experimental periodontitis was induced by applying an eight-shaped ligature to the cervical region of the lower incisor teeth while maintaining an open mouth with a speculum, following the induction of general anesthesia via intraperitoneal (IP) combination of ketamine (100 mg/kg) and xylazine (25 mg/kg). This ligature enhanced the production of plaque, provoked gingival irritants, and led to the development of periodontal disease. All through the 30-day experimental period, the ligature was checked every other day (Mustafa et al., 2022).

#### 2.1.2 Platelet-rich plasma (PRP) preparation

Through cardiac puncture, 9.0 mL of blood was extracted from the rats using a syringe fitted with an 18 G injection needle. The drawn blood was then put into a tube holding 1.0 mL of acid-citrate-dextrose solution, and it was centrifuged at 1500 rpm for 8 min. The platelets, leukocytes, and a small number of erythrocytes were then removed into a long cannula along with the buffy coat plasma, five minutes at 3000 rpm were dedicated to the second centrifugation. The lowest part of the plasma had platelet pellets accumulating, while the top layer of plasma had fewer platelets. About 1.0 mL of plasma containing platelet pellets was utilized as autologous PRP.

#### 2.1.3 Intra-gingival injection of PRP

A microsyringe (Hamilton Robotics, Switzerland) was used to inject ten microliters of platelet-rich plasma (PRP) into each rat after it had been given general anesthesia. Every injection had a volume of 10 µL, which was the maximum volume the tissue could tolerate without being repulsed. A single injection was administered every week for thirty days.

### 2.2 Obtaining dental specimens for histological assessment

Pentobarbitone (200 mg/kg, IP) was administered to rats on days 7 and 30 of the trial, 24 h following the prior local treatment. Using sterile tools, periodontal connective tissue samples were extracted from the teeth following the removal of the mandibles. Following a full day of preservation in a 10 % neutral buffer formalin, the samples were sliced in half on both sides posterior to the incisor teeth. They were also decalcified in HCl for a day and in 10 % EDTA solution for eight weeks at room temperature. The tissues were then managed using a standard paraffin embedding technique, which involved dehydrating, cleaning in xylene, and fixing in paraffin in the Histopathology Lab of Shoresh Hospital, Sulaimani Governorate. Three thin sections, each measuring 3 μm, were mounted on normal slides and stained with H&E, as well as for an IHC IL-1β and RANKL markers.

#### 2.2.1 Histopathologic examination

Two pathologists, blind to the prior treatment, examined each slide histologically using a light microscope at 10–400 magnification. The histology scores of bone healing were determined using the parameters listed in Table 1; the same lists were used for the evaluation and application of the histological score in this investigation (Mustafa et al., 2022).

**Table 1:** Assessment standards for histopathological bone healing scores (Mustafa et al., 2022).

Parameters	Scores	Interpretation
Re-epithelialization	0 1 2 3 4	Absence of epithelial proliferation Epidermal regeneration in 25–50 % of the tissue Epidermal regeneration in 75 % of the tissue Epithelial remodeling in 75 % of the tissue Full epidermal remodeling of the tissue
Granulation tissues	0 1 2 3 4	Immature and inflammatory reaction in 50–75 % Thin immature and inflammatory tissue in 25–50 % Thick mature and inflammatory tissue in 25–50 % Thick granulation and well-formed collagen matrix in 70 % Full tissue organization of the tissue
Inflammatory cells infiltration /section field	0 1 2 3 4	14–16 inflammatory cells 12–14 inflammatory cells 10–12 inflammatory cells 4–8 inflammatory cells 2–4 inflammatory cells
Angiogenesis/site	0 1 2 3 4	Absence of angiogenesis 1–2 newly formed vessels 4–6 newly formed vessels 6–8 newly formed vessels 8–10 newly formed vessels
Congestion, edema, and hemorrhage	0 1 2 3 4	Severe Moderate Moderate-mild Mild Absence

**Fig. 1:**
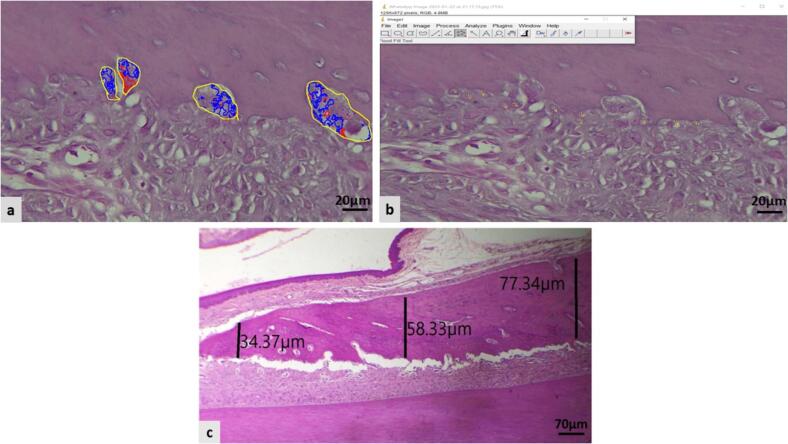
The alveolar bone section from rat’s incisor teeth: a and b: The measurement of osteoclast and osteoblast. c: The alveolar bone thickness measurement in three locations, (H&E stain).

By image J analyzer and Amscope^TM^ software in each section the bone cells including; osteoblasts and osteoclasts were counted from the alveolar bone surface (Fig. 1a,b). The alveolar bone thicknesses in the furcation area or crest, middle, and cervical sections were measured vertically concerning the cementum surface and the alveolar bone surface (Fig. 1c).

##### 2.2.1.1 Immunohistochemical analyses

At two durations, on days 7 and 30, the paraffinized ribbons of 4 µm thickness were deparaffinized by using xylene. Slides with paraffin-embedded were hydrated after being dewaxed in xylene. Antigen retrieval solution, a sodium citrate buffer (pH 6.5) was used for heating the sections in a microwave oven for 20 min, they were cooled in de-ionized water. To reduce endogenous peroxidase activity, the section was separated as well and covered with 3 % hydrogen peroxide for 12 min. The sections were incubated overnight with primary antibodies; IL-1β rabbit polyclonal antibody (1:600; Biorbyt, US) and RANKL rabbit polyclonal antibody (1:100; Biorbyt, US). Using streptavidin combined with horseradish peroxidase (Biorbyt, US) improved the reaction as directed by the supplier. To see the reaction, diaminobenzidine was used to observe the process's outcomes (Biorbyt, US). After counterstaining, hematoxylin was dried according to protocol and covered with coverslips.

**Fig. 2:**
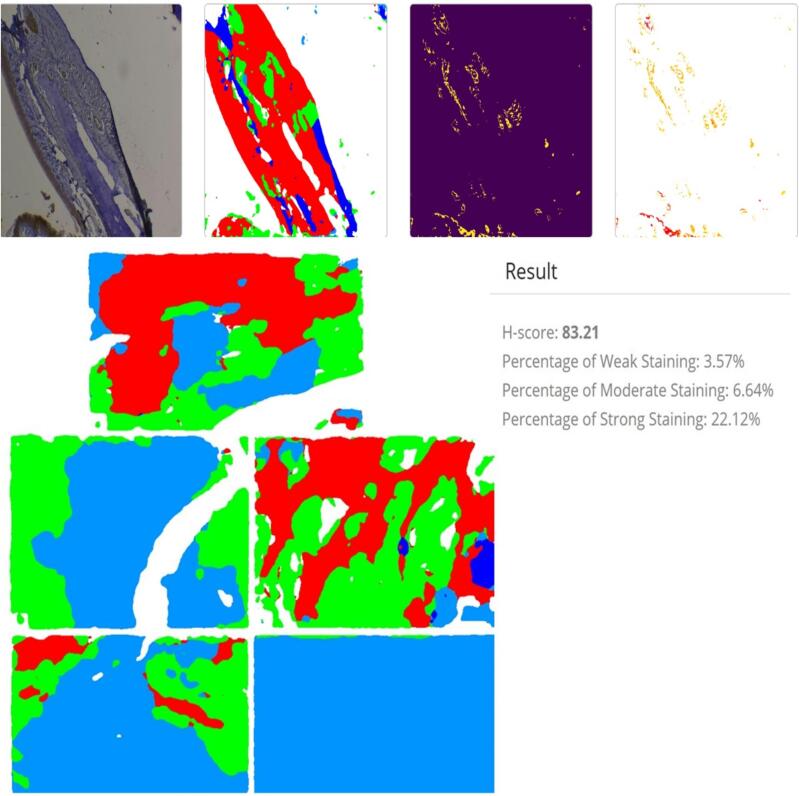
The IHC score by using AHSQ software for quantitative assessment of extensity and density of positive immune cells.

Image analysis software (AHSQ) was used to examine slices and evaluate the extensity and intensity of the positive immune cells (H score). The immunopositively cells showed; that the cytoplasm was stained with brownish granules of IL-1β and RANKL, the nuclei remaining blush color and not stained. The intensity of IL-1β and RANKL On a scale of weak (+1), moderate (+2), moderate-strong (+3), and strong (+4), staining was graded. In IHC staining of RANKL and IL-1β, respectively, the extent of positively stained bone cells, including osteocyte, osteoblast, osteoclast, endothelial cells, and inflammatory cells, was measured as no staining or (0) for 0–5 % positive staining, (1) for 6–20 % positive staining, (2) for 21–40 % positive staining, (3) for 41–65 % positive staining, and (4) for > 65 % positive staining. A positive reactivity extent was defined as a total staining score ranging from 0 to 16, and the staining intensity was amplified (Fig. 2).

### 2.3 Statistical analysis

SPSS was performed for statistical analysis of data. Shapiro-Wilk test was assessed for normality. ANOVA was undertaken for intergroup relations and repeated measures. For post hoc comparisons Bonferroni test was carried out. The confidence interval assumed was 95 %.

## 3 Results

### 3.1 The effect of PRP on improving the alveolar bone and periodontal ligament reconstruction

**Fig. 3:**
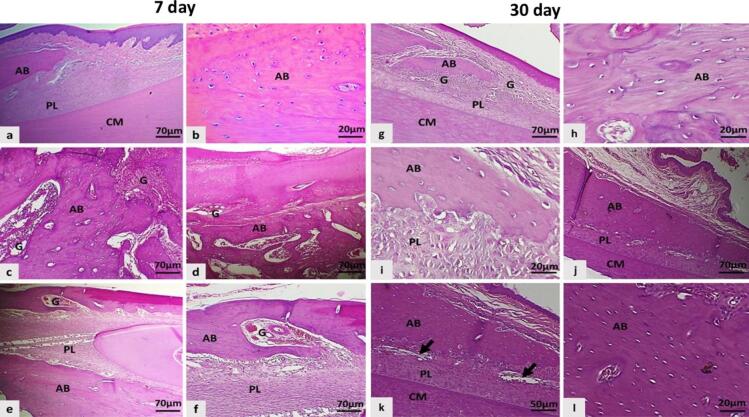
The microscopic section of periodontal tissue and an incisor tooth of a rat: a and b: Intact structures and organization of the periodontal tissue. c, and d: in the insertion point severe sloughing of gingival lining epithelium with granulation tissue was seen, as disorganized bone trabeculae, and a large gap between the periodontal ligament and cementum. e and f: Slight shedding of periodontal ligament and cementum, mild-moderate granulation tissue in the alveolar bone and the periodontal ligament in PRP treated group. g-i: A normal cementum surface is connected to a thin, uneven alveolar bone surface that has an osteoclast-containing resorption area, mild granulation tissue, and less organized proliferating periodontal ligament tissue in the control positive group. j-l e: Angiogenesis (black arrows) and regular cementum surface are connected to well-thick, proliferating periodontal ligament tissue that is well-organized and densely packed with alveolar bone, with a resorption area that contains few osteoclasts in PRP-treated group, (AB; alveolar bone, PL; periodontal ligament, CM; cementum, G; Granulation tissue), (H&E stain).

The microscopic periodontium tissues on day 7 in negative control revealed normal organization with intact histologic structures (Fig. 3a,b) vs. the control positive group which presented marked bone loss and damage including; sloughing of gingival lining epithelium and detached periodontal ligament (PL) from cementum, thin vascular granulation tissue in the gingival tissue (Fig. 3c,d). Also, the result in control positive presented irregular bone surface and unorganized bony trabeculae, absence of Sharpey's fibers vs. the rat group treated with PRP revealed bone trabeculae much more organized than those found in control positive groups, fibrovascular granulation tissue increasing fibroblast proliferation in PL but Sharpey's fibers were not produced yet (Fig. 3e,f).

By day 30, the periodontal tissue had returned to a normal histologic structure and PRP had improved bone repair, it showed typical remodeling (Fig. 3g-i), the alveolar bone more organized, intact periodontal ligament into a regular cementum surface (score 4) in comparison to the control positive group, which showed thick fibrovascular granulation tissue with less organized periodontal tissue attached to the regular cementum surface with a focal of Sharpey's fiber, and thin, uneven bone surface with bone loss in 75 % of cases, (Fig. 3j-l).

**Fig. 4:**
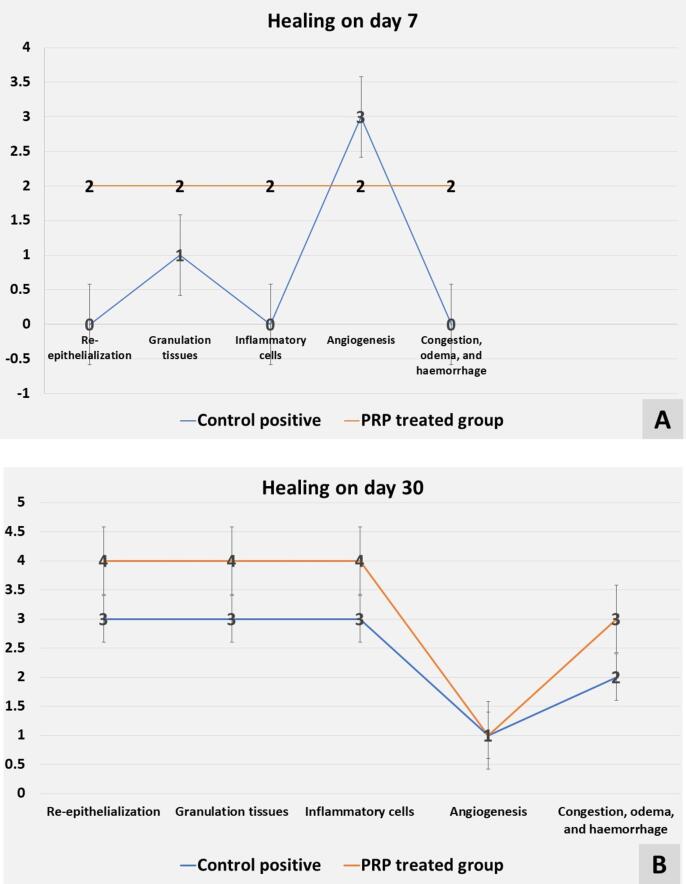
The line chart shows the histopathologic healing scores in the control positive and the PRP-treated groups on day 7 in (A) and on day 30 in (B).

The PRP enhanced the process of healing on days 7 and 30 documented by the scoring system as day 7 showed; a healing score regarded as (Score 2) for reepithelization in which only 75 % of the gingiva was repaired, thick granulation tissue in 40 % of the tissue (Score 2), inflammatory cells was score = 2 (11 inflammatory cells per section field), angiogenesis by score 2 (4 per section field), and mild-moderate edema and congestion (score = 2) vs. to the control positive group that had a delay in alveolar bone and PL healing by decreasing the score as; Score 0 for reepithelization in which none of the gingiva was repaired, thin granulation tissue in 40 % of the tissue (Score 1), inflammatory cells was score = 0 (14 per section field), angiogenesis by score 3 (7 per section field), and severe edema and congestion (score = 0) as in Fig. 4A.

Also on day 30 PRP effectively improved the process of repair by increasing the histological score degrees as follows; complete gingival remodeling (Score 4) with complete alveolar bone and PL tissue organization (Score 4), few inflammatory cells (3 per section field, score 4), angiogenesis (2 per section field, score = 1), and mild edema and congestion (Score 3) vs. to the control positive group in which there was a reduction in the score degree by; Moderate gingival remodeling by 70 % (Score 3) with thick granulation and well-formed mineralized collagen matrix in 70 % of the alveolar bone and PL tissue organization (Score 3), inflammatory cells (7 per section field, score 3), few angiogenesis (3 per section field, score 2), and moderate odema and congestion (Score 1) as seen in Fig. 4B.

### 3.2 The impact of PRP on alveolar bone mass formation and regulation of the osteoblast and the osteoclast proliferation

The PRP-treated group significantly attenuated the alveolar bone loss on days 7 and 30 respectively and enhanced the bone mass increasing vs. the control positive group that showed significant (P ≤ 0.05) bone loss (Table 2).

**Table 2:** The measurement of alveolar bone mass thickness in experiment groups.

Time	Negative Control	Positive Control	Treatment (PRP)
7	58.14 ± 0.1^a^	45.65 ± 6.04^b^	55.08 ± 12.54^c^
30	65.14 ± 1.54^a^	52.41 ± 6.12^b^	62.35 ± 2.18^c^

P ≤ 0.05 was used to determine the significance for each distinct alphabetical letter within each row, with values expressed by Mean ± SE.

Throughout the trial period, periodontitis increased bone turnover and osteoclast proliferation, peaking in the control positive group relative to the control negative and PRP-treated groups, while on days 7 and 30 the PRP in the treated rats group significantly (P ≤ 0.05) reduced the proliferation of the osteoclast and the bone resorption activity vs. control positive group, (Table 3). Also, the PRP facilitated or promoted the activity of osteoblast proliferation and increased the alveolar bone mass formation significantly (P ≤ 0.05) vs. the control positive group as in Table 3 in both duration day 7 and day 30.

**Table 3:** The counting of osteoblast and osteoclast numbers in studied groups.

Time	Negative Control	Positive Control	Treatment (PRP)
**7**	80.25 ± 1.20^a^	62.43 ± 0.80^b^	76.68 ± 0.24^c^
	0.00 ± 0.00^a^	2.23 ± 0.10^b^	1.00 ± 0.20b^b,c^
**30**	82.50 ± 1.20^a^	68.12 ± 0.20^b^	80.25 ± 0.22^c^
	0.00 ± 0.00^a^	4.43 ± 0.44^b^	1.55 ± 1.41^b,c^

P ≤ 0.05 was used to determine the significance for each distinct alphabetical letter within each row, with values expressed by Mean ± SE.

The osteoblast count is shown in the first row and the osteoclast count is shown in the second.

### 3.3 The effect of PRP in immunohistochemical expression of IL-1β and RANKL in periodontal tissue

**Fig. 5:**
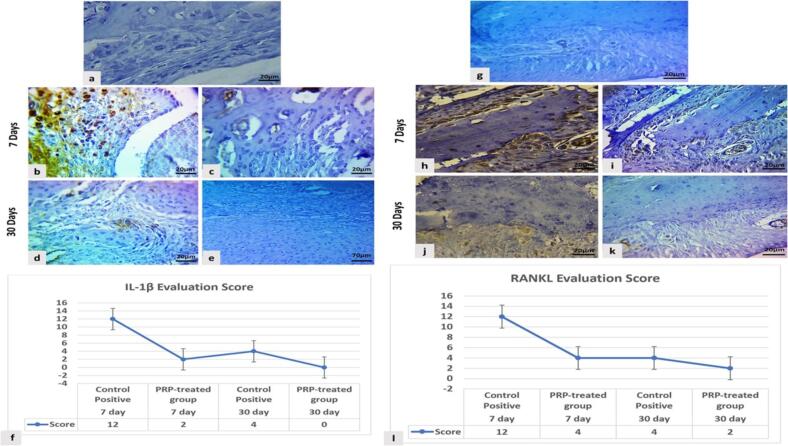
Immunohistochemical section periodontal tissue of cytoplasmic expression of IL-1β and RANKL among different studied groups showed; a: No positive stain (score = 0) in the control negative group. b: Strong diffuse positive staining (score = 12) in the control positive group. c: Moderate staining in endothelium only (score = 2) in the PRP group. d: Moderate positive cells in both inflammatory and endothelium (score = 4) in the control positive group. e: No staining (score = 0) in the PRP group. f: The score degree for IL-1β expression in the studied groups. g: No positive stain (score = 0) in the control negative group. h: Strong diffuse positive staining (score = 12) in the control positive group. i: Moderate staining in osteoclast only (score = 4) in the PRP group. j: Moderate positive cells in both osteocyte and osteoclast (score = 4) in the control positive group. k: Weak-focal staining (score = 2) in the PRP group. l: The score degree for RANKL expression in the studied groups.

Throughout the experimental study, the PRP on days 7 and 30 attenuated the inflammatory reaction of periodontitis by decreasing the expression of IL-1β in both the endothelium and inflammatory cells in the periodontal tissue. Also, PRP attenuated the expression of RANKL in bone cells. For example, regarding IL-1β on day 7, no positive cell was recorded (score = 0) in the control negative group in comparison to the control positive group showed diffuse expression with the strong staining in endothelial cell lining and inflammatory cells (score = 12), while in the PRP group decreased its expression and only moderate staining seen in endothelium (score = 2). For 30 days the moderate staining of positive cells was detected in both inflammatory cells and endothelium (score = 4) in the control positive group vs. in the PRP group showed no positive cells (score = 0), (Fig. 5a-f).

Regarding the RANKL expression the PRP plays an important role in reducing its expression in both durations, on day 7, there were no positive cells (score = 0) in the control negative group vs. the control positive group that showed strong and diffuse positive staining (score = 12), while PRP attenuated its expression to moderate staining only expressed in osteoclast (score = 4). On day 30, the control positive group revealed expression of moderate positive cells in both osteocyte and osteoclast (score = 4) vs. the PRP group RANKL expression was seen weakly in a few areas (score = 2), (Fig. 5g-l).

## 4 Discussion

One of the most common inflammatory diseases is periodontitis (Kuang et al., 2017). Many studies examining the various treatment modalities are being carried out; these vary based on the severity of the ailment. Earlier, every effort was directed towards pathogen removal, inflammation management, and patient immunity modification (Riccia et al., 2007).

It is clear from the current study that PRP increased bone repair and regeneration when compared to the periodontitis group, given that a range of cells, including mesenchymal stem cells, osteoblasts, and osteoclasts, are usually engaged in the development of bone tissue. When stem and progenitor cells are attracted to the defect location by the growth factors and platelets found in PRP, bone defects can be repaired much more quickly. PRP has particular immunomodulatory and antimicrobial properties, also it can control mesenchymal stem cells and bone cells (Wang et al., 2019). PRP's ability to stimulate bone formation by promoting osteoblastic differentiation has been the subject of numerous research; our study showed that PRP increased bone mass by growing osteoblast proliferation and declining osteoclast activity to have equilibrium between bone formation and bone lysis when compared to the control positive group that had bone loss due to maximum number of osteoclast cells vs. to the osteoblast cells, which is in consistence to the former studies of who documented the PRP-induce osteoblast proliferation (Rodriguez et al., 2014, Anitua et al., 2024). Additionally, PRP enhanced angiogenesis (score 2) in this study in comparison to the control positive (score 3), which is consistent with earlier findings showing that PRP has shown to be a powerful angiogenesis facilitator, encouraging the neovascularization that is essential for sustaining the regeneration processes involved in bone healing (Hu and Olsen, 2016, Du et al., 2018). VEGF, PDGF, Eselectin, and intercellular adhesion molecule1 (ICAM1) are angiogenic factors that are present in PRP and act as a primary driver of neovascularisation in endothelial cells and primary osteoblast coculture systems. PRP is the main force behind neovascularisation, which supplies nutrients and oxygen to the healing region to meet the metabolic needs of mending cells. In addition, it acts as a pathway for the migration of mesenchymal stem cells and osteoprogenitor cells, both of which are essential for the development of new bone tissue (Zhang et al., 2013, Zhang et al., 2018).

The components of the bacterial plaque and the host's defense mechanisms are two parts of the complex, multifactorial process that is periodontitis. Destroying the alveolar bone and underlying ligament can result from periodontitis inflammation. The new TNF receptor-related protein known as receptor activator of NF-KB ligand (RANKL) is crucial for the activation and differentiation of osteoclasts. It has been shown that periodontitis causes osteolysis by osteoclasts (Vernal et al., 2006, Nagasawa et al., 2007).

The balance between bone formation and resorption, which is reliant on the RANKL-RANKLOP Gaxis, is maintained by osteoblasts and osteoclasts. In the current study, the highest score of 12 was recorded in the control positive group compared to the PRP-treated group (score = 4), which utilized the RANKL as a marker of osteoclastogenesis activity. Additionally, the current result is supported by the findings of earlier research demonstrating that areas with destructive periodontal activity have higher levels of RANKL, because the RANKL is the NF-kB activation receptor present on osteoclast surfaces, and its receptor plays a crucial role in controlling the differentiation, recruitment, and function of osteoclasts, which RANKL is also involved in periodontal bone resorption (Ionel et al., 2015, Gibertoni et al., 2017). Because multiple investigations have verified the role of the Wnt pathway in PRP-mediated osteoclast differentiation inhibition. According to reports, a number of lignin-like substances suppress RANKL-induced osteoclastogenesis and prevent osteoclasts from mediating bone resorption (Goto et al., 2006). Previous findings corroborate our hypothesis that PRP could reduce the loss of alveolar bone in rats with ligature-induced periodontitis by promoting osteoblastic differentiation and reducing osteoclast activation via reducing the expression of RANKL, a molecule related to osteoclastogenesis (Wang et al., 2018, Elgamily and Denewar, 2021, Ceresa et al., 2024).

These alterations in RANKL expressions that induced resorption may have resulted from elevated levels of proinflammatory mediators, such as IL-1β, which we also found to be highly expressed in the control positive group compared to the PRP-treated groups in the current study. These results are consistent with earlier research that linked oxidative stress and its detrimental effects to periodontitis (Kose et al., 2016). Research has shown that PRP reduces inflammation by blocking IL-1β (Yin et al., 2016). Additional research revealed that IL-1 promotes osteoclastogenesis through two independent processes: OPG expression suppression, which is mediated by PGE2 production, and direct enhancement of RANKL expression (Suda et al., 2004). In vitro and in vivo osteoclastogenesis regulation, as well as IL-1 positive correlation with RANKL expression, have all been shown to be important aspects of the periodontal disease process (Wei et al., 2005, Kawai et al., 2006, Fujihara et al., 2014). Likewise, in additional research, there was a positive correlation found between the severity of periodontal disease and the values of the RANKL/OPG ratio (Branco-de-Almeida et al., 2017, Ochanji et al., 2017). In this study suggested that the possible inhibitor of osteoclastogenesis may be due to high content of PRP for T-GF-b.

## 5 Conclusion

PRP can enhance osteoblast proliferation and inhibit the osteoclast proliferation, as well as accelerate bone repair by downregulation of IL-1β and RANKL that can be involved in regulation of bone cells activity.

## 6 Author statements

The experiment was carried out by HHM and SMAH, supervised, came up with the initial concept. SMAH, HJM, and HKZ examined the data, produced the article, and double-checked the Histopathological usage and paper. The data analysed by AKM and MOM. The paper is also written by HHM, SMAH, and HFM. The work has been read and approved by all authors.

## Ethical statement

The Ethics Committee of the College of Veterinary Medicine at Sulaimani University in Kurdistan has approved all procedures involving the handling, care, and sampling of animals (permission 030526, 11 December 2023).

## Declaration of competing interest

The authors declare that they have no known competing financial interests or personal relationships that could have appeared to influence the work reported in this paper.
